# Epidemiology, Symptomatology, and Risk Factors for Long COVID Symptoms: Population-Based, Multicenter Study

**DOI:** 10.2196/42315

**Published:** 2023-03-07

**Authors:** Martin Chi-Sang Wong, Junjie Huang, Yuet-Yan Wong, Grace Lai-Hung Wong, Terry Cheuk-Fung Yip, Rachel Ngan-Yin Chan, Steven Wai-Ho Chau, Siew-Chien Ng, Yun-Kwok Wing, Francis Ka-Leung Chan

**Affiliations:** 1 Jockey Club School of Public Health & Primary Care The Chinese University of Hong Kong Sha Tin China; 2 Centre for Health Education and Health Promotion Faculty of Medicine The Chinese University of Hong Kong Sha Tin China; 3 School of Public Health Peking University Beijing China; 4 School of Public Health The Chinese Academy of Medical Sciences and Peking Union Medical College Beijing China; 5 Department of Medicine and Therapeutics Faculty of Medicine The Chinese University of Hong Kong Sha Tin China; 6 Medical Data Analytics Centre The Chinese University of Hong Kong Sha Tin China; 7 Institute of Digestive Disease Faculty of Medicine The Chinese University of Hong Kong Sha Tin China; 8 Department of Psychiatry Faculty of Medicine The Chinese University of Hong Kong Tai Po China

**Keywords:** COVID-19, epidemiology, symptom, risk factor, long COVID, multicenter survey, general population

## Abstract

**Background:**

Long COVID induces a substantial global burden of disease. The pathogenesis, complications, and epidemiological and clinical characteristics of patients with COVID-19 in the acute phase have been evaluated, while few studies have characterized the epidemiology, symptomatology, and risk factors of long COVID symptoms. Its characteristics among patients with COVID-19 in the general population remain unaddressed.

**Objective:**

We examined the prevalence of long COVID symptoms, its symptom patterns, and its risk factors in 4 major Chinese cities in order to fill the knowledge gap.

**Methods:**

We performed a population-based, multicenter survey using a representative sampling strategy via the Qualtrics platform in Beijing, Shanghai, Guangzhou, and Hong Kong in June 2022. We included 2712 community-dwelling patients with COVID-19 and measured the prevalence of long COVID symptoms defined by the World Health Organization (WHO), and their risk factors. The primary outcomes were the symptoms of long COVID, with various levels of impact. A descriptive analysis of the prevalence and distribution of long COVID symptoms according to disease severity was conducted. A sensitivity analysis of increasing the number of long COVID symptoms was also conducted. Univariate and multivariate regression analyses were performed to examine the risk factors of severe long COVID symptoms, including age, gender, marital status, current occupation, educational level, living status, smoking habits, monthly household income, self-perceived health status, the presence of chronic diseases, the use of chronic medication, COVID-19 vaccination status, and the severity of COVID-19.

**Results:**

The response rate was 63.6% (n=2712). The prevalence of long COVID, moderate or severe long COVID, and severe long COVID was 90.4% (n=2452), 62.4% (n=1692), and 31.0% (n=841), respectively. Fatigue (n=914, 33.7%), cough (n=865, 31.9%), sore throat (n=841, 31.0%), difficulty in concentrating (n=828, 30.5%), feeling of anxiety (n=817, 30.2%), myalgia (n=811, 29.9%), and arthralgia (n=811, 29.9%) were the most common severe long COVID symptoms. From multivariate regression analysis, female gender (adjusted odds ratio [aOR]=1.49, 95% CI 1.13-1.95); engagement in transportation, logistics, or the discipline workforce (aOR=2.52, 95% CI 1.58-4.03); living with domestic workers (aOR=2.37, 95% CI 1.39-4.03); smoking (aOR=1.55, 95% CI 1.17-2.05); poor or very poor self-perceived health status (aOR=15.4, 95% CI 7.88-30.00); ≥3 chronic diseases (aOR=2.71, 95% CI 1.54-4.79); chronic medication use (aOR=4.38, 95% CI 1.66-11.53); and critical severity of COVID-19 (aOR=1.52, 95% CI 1.07-2.15) were associated with severe long COVID. Prior vaccination with ≥2 doses of COVID-19 vaccines was a protective factor (aOR=0.35-0.22, 95% CI 0.08-0.90).

**Conclusions:**

We examined the prevalence of long COVID symptoms in 4 Chinese cities according to the severity of COVID-19. We also evaluated the pattern of long COVID symptoms and their risk factors. These findings may inform early identification of patients with COVID-19 at risk of long COVID and planning of rehabilitative services.

## Introduction

COVID-19 has posed an unprecedented challenge to public health worldwide. As of July 9, 2022, more than 551 million cases and 6.3 million deaths have been reported [1]. Apart from the acute phase of the infection, the disease may also manifest as persistent, lingering symptoms known as long COVID [2-5]. The World Health Organization (WHO) has defined long COVID as a condition that occurs in patients with COVID-19 usually 3 months from the onset of the disease, with symptoms that last for at least 2 months and that the symptoms could not be attributed to an alternative diagnosis [6]. These long-term symptoms could exert a devastating effect [7], as they could involve multiple organ systems, and have been linked to psychosocial consequences [8]. A study has found that almost 10% of people with COVID-19 reported long COVID stmptoms 4-12 weeks after the infection [9,10].

The impact of long COVID symptoms has been observed even in patients with mild COVID-19, patients who do not require respiratory support or intensive care, moderate-to-severe cases among hospitalized patients who turn out to have cleared SARS-CoV-2 and have been discharged from hospitals, mild-to-moderate cases in outpatient clinics, and even children [11]. It has been reported that a substantial proportion of patients with COVID-19 continue to suffer from long-term adverse effects involving almost all bodily systems, including respiratory, gastrointestinal, neuropsychiatric, cardiovascular, and dermatological systems [12]. A recent study performed by our team showed that approximately 76% of patients with COVID-19 suffered from long COVID symptoms, including fatigue, poor memory, and hair loss, within 6 months of hospital admission [13].

The prevalence of long COVID symptoms appeared not only in Hong Kong and other Chinese cities but also across different countries [14]. For example, a study in Germany described different symptoms of long COVID, including headache, cough, shortness of breath, fatigue, dysosmia or anosmia, brain fog, or cognitive impairment [15]. Similar to these studies, the pattern of long COVID symptoms and their impacts have been investigated. For example, fatigue was the most common symptom in patients who had long COVID irrespective of the severity of their initial infection. Patients who had severe fatigue between 3 and 6 months might not encounter symptom improvement or resolution in the long term. Even those patients who had mild COVID-19 could develop long-term symptoms, including cough, fatigue, low-grade fever, shortness of breath, headaches, chest pain, muscle pains and weakness, metabolic disruption, neurocognitive difficulties, and gastrointestinal upset [3]. Long COVID symptoms could largely affect health-related quality of life and activities of daily living [16,17].

There could be many contributing factors that lead to prolonged patient recovery, such as deconditioning, relapse or reinfection, weak or absent antibody response, inflammatory and other immune reactions, and posttraumatic stress [3]. Risk factors that are associated with sociodemographic characteristics are important. These include gender, employment status, marital status, religion, multimorbidity, and living status [18-21].

Although the pathogenesis, complications, and epidemiological and clinical characteristics of patients with COVID-19 in the acute phase have been evaluated [22,23], few studies have characterized the epidemiology, symptomatology, and risk factors of long COVID. Previous studies examining these attributes are relatively few, mainly focused on hospitalized patients, or used a convenience sampling strategy [8]. In addition, the timing of assessment varied from 14 days to 6 months—or was not even reported—which is not consistent with the time frame of long COVID proposed by WHO, which is 3 months after COVID-19 infection [6]. In a recent review by Crook et al [8], 1 of the top priorities of research includes the precision epidemiology and risk factors of long COVID.

Hence, we aimed to fill these knowledge gaps by evaluating the prevalence of long COVID symptoms in 4 major Chinese cities using a representative sampling methodology. We also examined the distribution of COVID-19 symptoms and explored the risk factors of long COVID symptoms. We hypothesized that age, gender, the presence of chronic diseases, the use of chronic medications, the severity of COVID-19, and not receiving COVID-19 vaccines are associated with the occurrence of long COVID symptoms based on findings from recent studies [18,24,25].

## Methods

### Study Design

This was a cross-sectional survey of patients with COVID-19, aged ≥18 years, and residing in 4 cities of China: Beijing, Shanghai, Guangzhou, and Hong Kong. The study was launched on June 2, 2022, and closed on June 28, 2022. We used Qualtrics [26], an online survey platform. Inclusion criteria included age ≥18 years, with a diagnosis of COVID-19 from January 2020 to June 2022 in Beijing, Shanghai, Guangzhou, or Hong Kong. We excluded subjects without a history of COVID-19 infection. The survey collected the dates of COVID-19 vaccination, COVID-19 diagnosis, and recovery.

### Ethical Considerations

The Survey and Behavioural Research Ethics Committee of the Chinese University of Hong Kong approved the study (approval no. SBRE-21-0730). All eligible participants provided digital informed consent via electronic means at the beginning of the survey. The survey did not collect personally identifiable information, and we encrypted email addresses collected for survey distribution as anonymized respondent identities. All respondents’ data were recorded in an anonymous manner with a unique internally generated reference key. We assured the respondents of the anonymous, confidential nature of the study, where only aggregate data would be reported. We strictly adhered to the data privacy policy in the approved study protocol.

### Survey Instrument

An expert panel consisting of epidemiologists, physicians, public health professionals, professors, and biostatisticians composed and validated the survey. The survey was pilot-tested to optimize coherence and clarity and was subsequently revised according to the pilot findings. During the curation of survey items, we referred to published studies that used validated survey instruments for long COVID symptoms [13,27]. The survey comprised 31 questions and required a median of 13 minutes to complete. The information sheet of the survey provided background information about COVID-19. The survey was delivered in traditional Chinese for participants in Hong Kong and simplified Chinese for respondents in the other 3 cities of mainland China. Before commencement of the survey, prospective participants confirmed that they were aged 18 years or older and had previously been diagnosed with COVID-19.

The survey collected respondents’ sociodemographic information, past medical history, use of chronic medications, history of COVID-19 vaccines received, and severity of COVID-19. The survey contained several parts: First, we collected the respondents’ sociodemographic information, including gender, marital status, employment status, types of jobs, educational level, living status, smoking habits, income level, self-reported health status, and long-term medication. In addition, we asked for the dates of positive and negative COVID-19 tests, the severity of COVID-19, the number of COVID-19 vaccines that were received, and the dates of taking vaccination. To identify the severity of COVID-19, we used the COVID-19 WHO severity classification [28] to assign respondents to 4 categories (mild, moderate, severe, and critical) based on the presence of pneumonia (eg, adults with clinical signs of pneumonia, respiratory rate>30 breaths/minute, severe respiratory distress, or SpO_2_<90% on room air; WHO mentioned that a diagnosis can be made on clinical grounds with the assistance of chest imaging, such as radiograph, computed tomography (CT) scan, or ultrasound), hypoxia requiring oxygen, the need for mechanical ventilation, and a history of admission to the intensive care unit (ICU) due to COVID-19.

The respondents’ medical history before and after the diagnosis of COVID-19 was also collected. Respondents were asked whether they had certain types of diseases before diagnosis (yes or no), the year of disease diagnosis, whether they took medications for the diseases (yes or no), and an open-ended field allowing them to document their medications. Medical conditions on the survey were infection, tumor, metabolic diseases, hematological disorders, mental illness, nervous system disease, circulatory system disease, respiratory disease, digestive disease, reproductive and urinary diseases, pregnancy complications, immune system disease, skin and subcutaneous tissue diseases, and musculoskeletal and connective tissue disease, which have been used in previous studies on the association between comorbidities and COVID-19 infection [29].

We also inquired about the presence of COVID-19 symptoms encountered by the respondents that were persistent for at least 3 months after COVID-19 infection. Referring to previous surveys [13,27], we listed 30 common symptoms: fatigue, fever, chills, inability to perform exercise, night sweats, hair loss, headache, dizziness, chest pain, rapid heartbeat, cough, sputum, sore throat, runny nose, dyspnea, arthralgia, myalgia, nausea, vomiting, diarrhea, abdominal pain, stomachache, anosmia, loss of taste, blurred vision, difficulty in concentration, difficulty in fall into asleep, feeling anxious, feeling sad, and memory problems. An open-ended field allowed the respondents to document other symptoms. These symptoms also appeared in “Symptoms of Infection with Coronavirus-19 (SIC),” which is a comprehensive patient-reported outcome measure developed to evaluate vaccines and treatment for COVID-19 [30]. To capture clinically meaningful symptoms, we assigned a 4-point Likert scale to assess the impact of the symptoms on the respondents’ activities of daily living: 1 (no impact), 2 (little impact), 3 (moderate impact), and 4 (high impact). In addition, we classified the symptoms according to the bodily systems involved.

### Sampling Strategy

The sample partners of Qualtrics randomly selected eligible respondents for the study, aiming for national representation through routers and a sophisticated application programming interface (API). To enhance the representativeness of the survey, we used a simple random sampling strategy to recruit potential participants by trying to match population demographics to our survey. The study collected data on the population of each city, and eligible participants were randomly selected and invited to complete the survey. The survey was accessed through the website, a smartphone, or other e-devices. Data scrubbing was subsequently performed after receiving all survey responses to remove unfavorable data, optimizing data accuracy and reliability. A more detailed survey methodology has been described elsewhere [26].

### Outcome Variables and Sample Size

The primary outcome variable was the prevalence of long COVID symptoms, moderate or severe long COVID symptoms, and severe long COVID symptoms. We defined these 3 outcome variables as having at least 1 symptom with any degree of impact, at least 1 symptom with moderate or severe impact, and at least 1 symptom with severe impact, respectively. The secondary outcomes included the distribution of long COVID symptoms and their patterns. We assumed the proportion of the primary outcome as 50%, which provided a maximum sample size for each group. From the formula precision=1.96 × √[(p) × (1 – p)/N], where "p" refers to proportion of the primary outcome, a sample size of approximately 550 respondents would achieve a precision level of 0.04 for each city, so a total of 2200 respondents were required.

### Statistical Analysis

We conducted a descriptive analysis of the prevalence and distribution of long COVID symptoms according to their severity. As the prevalence of self-reported long COVID symptoms was high, we also conducted a sensitivity analysis of increasing the number of long COVID symptoms. The proportion of long COVID symptoms, moderate or severe long COVID symptoms, and severe long COVID symptoms was computed for each city. To examine the risk factors of severe long COVID symptoms, we performed univariate and multivariate regression analyses with the primary outcome as a binary variable. The covariates included age, gender, marital status, current occupation, educational level, living status, smoking habits, monthly household income, self-perceived health status, the presence of chronic diseases, the use of chronic medication, COVID-19 vaccination status, and the severity of COVID-19. We classified the respondents’ occupations based on the recommendation by the Environmental Modelling Group (EMG) [31]. We entered all covariates with *P*<.25 in univariate analysis into the regression model and evaluated their statistical significance defined as *P*<.05. We tested for variable interaction and multicollinearity in the regression analysis.

## Results

### Participant Characteristics

We received 7161 total entries and screened out 3862 (53.9%) surveys due to ineligibility. A total of 830 (11.6%) surveys were overquota, and quality control was performed on 248 (3.5%) surveys. There were 4024 (56.2%) complete surveys, and 1312 (32.6%) were removed by data scrubbing. The total sample size achieved was 2712 (37.9%), with a response rate of 63.6%. The number of respondents from Beijing, Shanghai, Guangzhou, and Hong Kong was 725 (26.7%), 718 (26.5%), 715 (26.4%), and 554 (20.4%), respectively. Among them, the majority (n=2182, 80.5%) were aged 25-44 years, female (n=1626, 60.0%), married (n=2422, 89.3%), engaged in full-time jobs (n=2612, 96.3%), and with a tertiary or higher educational level (n=2547, 93.9%). Most respondents engaged in occupations at high risk for COVID-19, including the catering industry (n=533, 19.7%), followed by transportation, logistics, and the discipline workforce (n=344, 12.7%) and the health care sector (n=201, 7.4%) [18]. Most were living with their spouse (n=2400, 88.5%) or children (n=2230, 82.2%), were nonsmokers (n=1817, 67.0%), and were approximately equally distributed according to their monthly household income in the strata HK $10,000 to ≥$60,000 (US $1275.04-$7650.22). The majority perceived their health as good (n=994, 36.7%) or average (n=952, 35.1%), followed by very good (n=466, 17.2%), poor (n=256, 9.4%), and very poor (n=44, 1.6%). Around 2515 (92.7%) respondents did not use chronic medications, and 335 (12.3%) reported at least 2 chronic conditions before COVID-19 diagnosis (Table 1).

**Table 1:** Respondent characteristics (N=2712).

Characteristics		Respondents, n (%)
**Age (years)**		
	18-24	139 (5.1)
	25-34	1150 (42.4)
	35-44	1032 (38.1)
	45-54	302 (11.1)
	55-64	71 (2.6)
	≥65	18 (0.7)
**Gender**		
	Male	1086 (10.2)
	Female	1626 (60.0)
**Marital status**		
	Single	277 (10.2)
	Married	2422 (89.3)
	Divorced	10 (0.4)
	Widowed	3 (0.1)
**Job status**		
	Full-time	2612 (96.3)
	Part-time	52 (1.9)
	Retired	19 (0.7)
	Housewife	16 (0.6)
	Others	13 (0.5)
**Current occupation: higher-exposure group**		
	Catering industry	533 (19.7)
	Health care sector	201 (7.4)
	Transportation, logistics, discipline workforce	344 (12.7)
**Current occupation: lower-exposure group**		
	Government or community	6 (0.2)
	Finance/insurance/banking	53 (2.0)
	IT/construction/manufacturing	189 (7.0)
	Student	94 (3.5)
	Unemployed/retired	177 (6.5)
	Housewife	74 (2.7)
**Educational level**		
	Primary or below	11 (0.4)
	Secondary	154 (5.7)
	Tertiary or above	2547 (93.9)
**Living with^a^**		
	Children	2230 (82.2)
	Spouse	2400 (88.5)
	Domestic worker	44 (1.6)
	Single	111 (4.1)
	Others	180 (6.6)
**Smoking status**		
	Nonsmoker	1817 (67.0)
	Ex-smoker quitted for ≥1 year	709 (26.1)
	Smoker	186 (6.9)
**Monthly household income (HK $/US $^b^)**		
	<10,000/<1275.04	128 (4.7)
	10,000-19,999/1275.04-2549.95	584 (21.5)
	20,000-29,999/2550.07-3824.98	747 (27.5)
	30,000-59,999/3825.11-7650.09	697 (25.7)
	≥60,000/≥7650.22	542 (20.0)
	Receiving a living allowance	6 (0.2)
	Refused to answer	8 (0.3)
**Self-perceived health status**		
	Very good	466 (17.2)
	Good	994 (36.7)
	Average	952 (35.1)
	Poor	256 (9.4)
	Very poor	44 (1.6)
**Chronic medication use**		
	No	2515 (92.7)
	Yes	197 (7.3)
**Number of chronic conditions before COVID-19 confirmed**		
	0	1988 (73.3)
	1	389 (14.3)
	2	90 (3.3)
	≥3	245 (9.0)

^a^The total proportion was more than 100% as we allowed multiple responses.

^b^HK $1=US $0.13.

### Severity of COVID-19

Most respondents suffered from pneumonia at the time of COVID-19 diagnosis (n=2141, 78.9%), required hospital admission for COVID-19 management (n=1944, 71.7%), needed oxygen due to COVID-19 (n=1427, 52.6%), and received antiviral agents (n=1747, 64.4%); see Multimedia Appendix 1, Table S1. A significant proportion necessitated artificial ventilation (n=986, 36.4%) and admission to the ICU (n=940, 34.7%). Up to 1552 (57.2%) of them had severe or critical COVID-19, and 1160 (42.8%) had mild or moderate severity of COVID-19. Most of the respondents had received vaccination before COVID-19 diagnosis, with the majority having taken 3 or more doses of Coronavac-Sinovac (n=1525, 56.2%), following by those who had taken 2 doses of Coronavac-Sinovac (n=786, 29.0%). There were about 169 (6.2%) respondents who had taken 2 or more doses of Pfizer-BioNTech.

### Long COVID Symptoms

**Figure 1:**
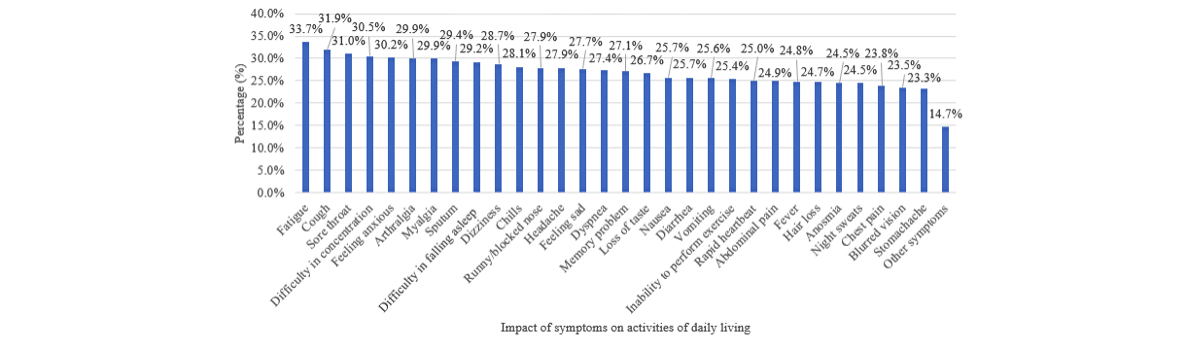
Distribution of long COVID symptoms. The highest proportion of respondents suffered from fatigue (n=914, 33.7%), cough (n=865, 31.9%), sore throat (n=841, 31.0%), difficulty in concentrating (n=828, 30.5%), feeling of anxiety (n=817, 30.2%), myalgia (n=811, 29.9%), arthralgia (n=811, 29.9%), sputum production (n=789, 29.4%), and difficulty in falling asleep (n=792, 29.2%).

**Figure 2:**
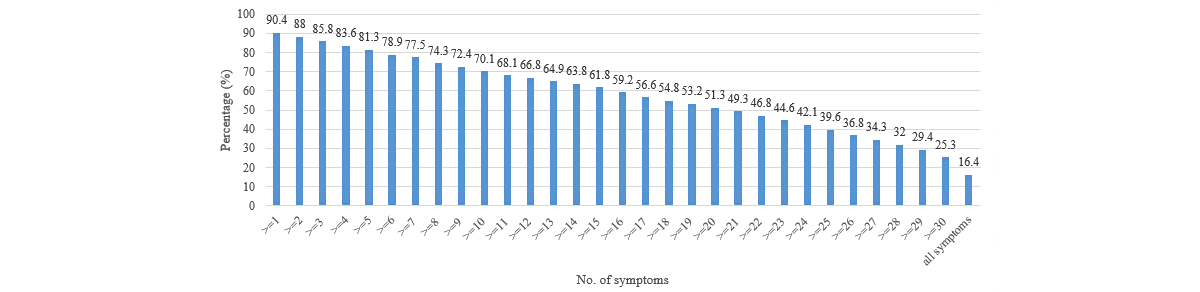
The prevalence of having long COVID symptoms of any severity, moderate or high severity, and high severity was 90.4% (n=2452), 62.4% (n=1692), and 31.0% (n=841), respectively. The symptoms were qualified in terms of self-perceived severity, and sensitivity analysis of using more than 1 symptom showed a better estimate of long COVID syndrome/disorder.

**Figure 3:**
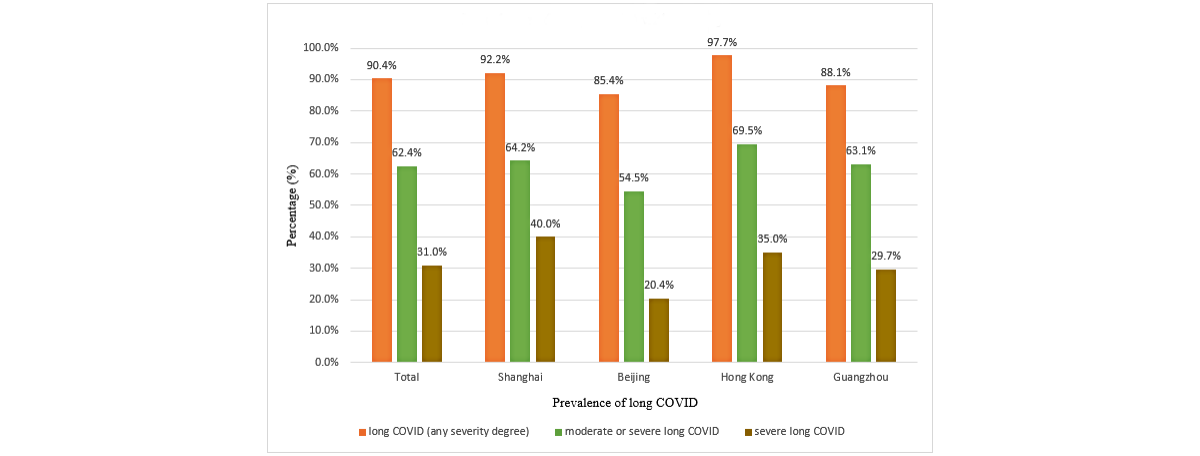
Prevalence of long COVID with severity. These prevalence figures were the highest in Hong Kong (n=541, 97.7%, n=385, 69.5%, and n=194, 35.0%, respectively) and Shanghai (n=662, 92.2%, n=461, 64.2%, and n=287, 40.0%, respectively), followed by Guangzhou (n=630, 88.1%, n=451, 63.1%, and n=212, 29.7%, respectively) and Beijing (n=619, 85.4%, n=395, 54.5%, and n=148, 20.4%, respectively).

Multimedia Appendix 1, Table S2, shows the distribution of long COVID symptoms. The highest proportion of respondents suffered from fatigue (n=914, 33.7%), cough (n=865, 31.9%), sore throat (n=841, 31.0%), difficulty in concentrating (n=828, 30.5%), feeling of anxiety (n=817, 30.2%), myalgia (n=811, 29.9%), arthralgia (n=811, 29.9%), sputum production (n=789, 29.4%), and difficulty in falling asleep (n=792, 29.2%); see Figure 1. The prevalence of having long COVID symptoms of any severity, moderate or high severity, and high severity was 90.4% (n=2452), 62.4% (n=1692), and 31.0% (n=841), respectively. The symptoms were qualified in terms of self-perceived severity, and sensitivity analysis of using 2 or more symptoms showed a better estimate of long COVID syndrome/disorder (Figure 2). These prevalence figures were the highest in Hong Kong (n=541, 97.7%, n=385, 69.5%, and n=194, 35.0%, respectively) and Shanghai (n=662, 92.2%, n=461, 64.2%, and n=287, 40.0%, respectively), followed by Guangzhou (n=630, 88.1%, n=451, 63.1%, and n=212, 29.7%, respectively) and Beijing (n=619, 85.4%, n=395, 54.5%, and n=148, 20.4%, respectively); see Figure 3. The prevalence figures were the highest in the second wave (from July to December 2020; n=67, 91.8%, n=48, 65.8%, and n=16, 21.9%, respectively) and the third wave (from January to June 2021; n=453, 91.7%, n=329, 66.6%, and n=175, 35.4%, respectively), followed by the first wave (from January to June 2020; n=122, 90.4%, n=64, 47.4%, and n=36, 26.7%, respectively), the fifth wave (from January to June 2022; n=1446, 90.1%, n=998, 62.2%, and n=503, 31.3%, respectively), and the fourth wave (from July to December 2021; n=364, 89.9%, n=253, 62.5%, and n=111, 27.4%, respectively); see Multimedia Appendix 1, Figure S1. A slightly negative correlation was found between the timing of COVID-19 and the number of long COVID symptoms (*β*=–.046, *P*=.016), supporting that long COVID symptom numbers decline with a longer duration of follow-up.

### Factors Associated With Long COVID Symptoms of Moderate-to-High Severity

From multivariate regression analysis (Table 2), female subjects (adjusted odds ratio [aOR]=1.49, 95% CI 1.13-1.95); patients engaged in transportation, logistics, or the discipline workforce (aOR=2.52, 95% CI 1.58-4.03); patients living with domestic workers (aOR=2.37, 95% CI 1.39-4.03); smokers/ex-smokers (aOR=1.55, 95% CI 1.17-2.05); respondents with poor or very poor self-perceived health status (aOR=15.4, 95% CI 7.88-30.00); those with more chronic diseases (1 chronic disease: aOR=1.92, 95% CI 1.24-2.97; 2 chronic diseases: aOR=2.71, 95% CI 1.21-6.05; ≥3 chronic diseases: aOR=2.71, 95% CI 1.54-4.79); chronic medication use (aOR=4.38, 95% CI 1.66-11.53); and critical severity of COVID-19 (aOR=1.52, 95% CI 1.07-2.15) were associated with the development of severe long COVID. Prior COVID-19 vaccination with Coronavac-Sinovac (aOR=0.35, 95% CI 0.14-0.90, *P*=.03) or Pfizer-BioNTech (aOR=0.22, 95% CI 0.08-0.63, *P*=.005) for at least 2 doses was a protective factor.

**Table 2:** Risk factors of severe long COVID (N=1030).

Characteristics		Respondents, n (%)	cOR^a^ (95% CI)	*P* value	aOR^b^ (95% CI)	*P* value
**Age (years)**						
	35-44	417 (60.5)	1 (reference)	.167	1 (reference)	.337
	18-24	57 (71.3)	1.62 (0.97-2.69)	.064	1.15 (0.59-2.22)	.686
	25-34	372 (58.5)	0.92 (0.74-1.14)	.452	1.09 (0.84-1.42)	.508
	45-54	138 (63.6)	1.14 (0.83-1.56)	.418	0.71 (0.47-1.08)	.108
	≥55	46 (65.7)	1.25 (0.75-2.10)	.397	1.20 (0.65-2.20)	.567
**Gender**						
	Male	357 (57.3)	1 (reference)	N/A^c^	1 (reference)	N/A
	Female	673 (63.0)	1.27 (1.04-1.55)	.022	1.49 (1.13-1.95)	.004
**Marital status**						
	Married	925 (60.8)	1 (reference)	N/A	N/A	N/A
	Single/divorced/widowed	105 (61.8)	1.04 (0.75-1.44)	.802	N/A	N/A
**Current occupation: higher-exposure group**						
	Catering industry	206 (63.4)	1 (reference)	<.001	1 (reference)	<.001
	Health care sector	63 (59.4)	0.85 (0.54-1.33)	.466	0.98 (0.58-1.65)	.941
	Transportation, logistics, discipline workforce	205 (84.0)	3.04 (2.01-4.58)	<.001	2.52 (1.58-4.03)	<.001
**Current occupation: lower-exposure group**						
	Government or community	3 (60.0)	0.87 (0.14-5.26)	.876	0.39 (0.04-3.52)	.405
	Finance/insurance/banking	18 (64.3)	1.04 (0.46-2.33)	.924	1.32 (0.54-3.23)	.542
	IT/construction/manufacturing	44 (50.0)	0.58 (0.36-0.93)	.024	0.36 (0.20-0.64)	.001
	Student	27 (67.5)	1.20 (0.60-2.41)	.610	1.10 (0.45-2.70)	.831
	Unemployed/retired	56 (61.5)	0.92 (0.57-1.49)	.747	0.60 (0.34-1.06)	.078
	Housewife	30 (63.8)	1.02 (0.54-1.93)	.953	1.07 (0.52-2.19)	.851
**Educational level**						
	Secondary or below	73 (70.2)	1 (reference)	N/A	1 (reference)	N/A
	Tertiary or above	957 (60.3)	0.64 (0.42-0.99)	.046	0.62 (0.37-1.05)	.073
**Living status**						
	Children	856 (61.2)	1.09 (0.84-1.41)	.513	1 (reference)	N/A
	Spouse	916 (60.9)	1.00 (0.73-1.36)	.979	N/A	N/A
	Single	63 (56.8)	0.83 (0.56-1.23)	.358	N/A	N/A
	Domestic workers/others	70 (70.0)	1.54 (0.99-2.38)	.055	2.37 (1.39-4.03)	.001
**Smoking status**						
	Nonsmoker	618 (55.0)	1 (reference)	N/A	1 (reference)	N/A
	Smoker	412 (72.5)	2.16 (1.74-2.69)	<.001	1.55 (1.17-2.05)	.002
**Monthly household income (HK $/US $^d^)**						
	<10,000/<1275.04 or Comprehensive Social Security Assistance (CSSA)/Disability Allowance (DA)	44 (69.8)	1 (reference)	.011	1 (reference)	.060
	10,000-19,999/1275.04-2549.95	218 (56.6)	0.56 (0.32-1.00)	.051	0.92 (0.47-1.82)	.811
	20,000-29,999/2550.07-3824.98	264 (56.8)	0.57 (0.32-1.00)	.051	1.10 (0.55-2.17)	.790
	30,000-59,999/3825.11-7650.09	244 (64.7)	0.79 (0.44-1.41)	.430	1.55 (0.78-3.11)	.214
	≥60,000/≥7650.22	260 (64.7)	0.79 (0.44-1.41)	.424	1.25 (0.62-2.53)	.526
**Self-perceived health status**						
	Very good	109 (39.6)	1 (reference)	<.001	1 (reference)	<.001
	Good	279 (46.3)	1.32 (0.98-1.76)	.064	1.51 (1.09-2.09)	.013
	Average	467 (74.5)	4.45 (3.29-6.01)	<.001	5.06 (3.58-7.13)	<.001
	Poor/very poor	175 (93.1)	20.5 (11.1-37.9)	<.001	15.4 (7.88-30.0)	<.001
**Presence of chronic diseases**						
	0	711 (54.5)	1 (reference)	<.001	1 (reference)	<.001
	1	117 (74.5)	2.44 (1.68-3.55)	<.001	1.92 (1.24-2.97)	.003
	2	37 (80.4)	3.43 (1.64-7.16)	.001	2.71 (1.21-6.05)	.015
	≥3	165 (89.2)	6.88 (4.27-11.08)	<.001	2.71 (1.54-4.79)	.001
**Chronic medication use**						
	No	955 (59.2)	1 (reference)	N/A	1 (reference)	N/A
	Yes	75 (93.8)	10.3 (4.15-25.7)	<.001	4.38 (1.66-11.5)	.003
**Vaccination status**						
	No vaccine taken	30 (83.3)	1 (reference)	.195	1 (reference)	.073
	Coronavac-Sinovac, 1 dose	40 (63.5)	0.35 (0.13-0.96)	.042	0.36 (0.12-1.11)	.075
	Coronavac-Sinovac, ≥2 doses	877 (60.4)	0.31 (0.13-0.74)	.008	0.35 (0.14-0.90)	.030
	Pfizer-BioNTech, 1 dose	3 (60.0)	0.30 (0.04-2.20)	.236	0.17 (0.02-1.77)	.139
	Pfizer-BioNTech, ≥2 doses	59 (59.0)	0.29 (0.11-0.75)	.011	0.22 (0.08-0.63)	.005
	Others	21 (58.3)	0.28 (0.09-0.84)	.023	0.22 (0.07-0.76)	.016
**Severity of COVID-19**						
	Mild	134 (47.9)	1 (reference)	<.001	1 (reference)	.009
	Moderate	286 (54.1)	1.28 (0.96-1.71)	.093	0.96 (0.69-1.34)	.811
	Severe	191 (66.3)	2.15 (1.53-3.01)	<.001	1.34 (0.90-1.98)	.150
	Critical	419 (70.4)	2.59 (1.93-3.48)	<.001	1.52 (1.07-2.15)	.018

^a^cOR: crude odds ratio.

^b^aOR: adjusted odds ratio.

^c^N/A: not applicable.

^d^HK $1=US $0.13.

## Discussion

### Principal Findings

This multicenter survey involving 4 Chinese cities showed that the prevalence of long COVID symptoms was high for symptoms of any severity, moderate or high severity, and high severity. We reported the most common long COVID symptoms and their distribution, as well as the risk factors of long COVID symptoms. Notably, COVID-19 vaccination was associated with a protective effect on the development of long COVID symptoms.

The prevalence of long COVID symptoms was high across different cities. Several studies have reported different incidence rates of long COVID symptoms at different observation periods, including 76% of patients at 6 months [5], 32.6%-87.0% of patients at 60 days [32,33], and 96% of patients at 90 days [34]. A study conducted in the Chinese city of Wuhan included 2469 patients who had confirmed COVID-19 and were discharged from the hospital between January and May 2020. Those who required high-flow nasal cannula (HFNC), noninvasive mechanical ventilation (NIV), or invasive mechanical ventilation (IMV) were more likely to suffer from long COVID symptoms and complications that affected their quality of life, including limitations in their mobility, usual activities, and mental health [14]. These findings were compatible with our results in which patients with critical severity of COVID-19 were more likely to have severe long COVID symptoms. The prevalence of long COVID symptoms was also common in other countries. For instance, a study found that 62% of 89 interviewed patients in the United Kingdom had long COVID symptoms for over 3 months, 52% for 6 months, and 49% for 9 months after their hospital admission [35]. In France, 51% of patients presented with at least 1 symptom after their COVID-19 diagnosis. Regarding severity, 20% of the patients required ICU care, including IMV, vasopressors, and extracorporeal membrane oxygenation during COVID-19 [31]. By using the same WHO definition of COVID-19 severity, a study in Turkey reported 47.5% of the participants suffering from 1 or more persistent symptoms, including outpatient and inpatient clinics, irrespective of disease severity. Most studies have reported high prevalence rates of long COVID symptoms, including Spain, Bangladesh, the United Kingdom, the United States, Nigeria, and Denmark [5].

In addition, research on the prevalence of long COVID symptoms has begun. Apart from respiratory symptoms, fatigue and neuropsychiatric symptoms have been the most frequently reported manifestations of long COVID. The UK Office for National Statistics (ONS) estimated the 5-week prevalence of fatigue to be 11.9% among patients with COVID-19 [36]. One cross-sectional study concluded that 92.9% and 93.5% of hospitalized and nonhospitalized patients with COVID-19, respectively, suffered from ongoing fatigue for 79 days following illness onset [17]. The exact mechanism of its appearance after COVID-19 is currently speculative. This has been attributed to dysfunctional inflammatory response pathways [37]. In addition, a repertoire of central, peripheral, and psychological factors might play a role [8], including congestion of the glymphatic system, hypometabolism in the frontal lobe and cerebellum, and direct SARS-CoV-2 infection of skeletal muscle fibres and neuromuscular junctions [8]. With regard to neuropsychiatric symptoms, the underlying mechanism may be related to glial cell activation, which damages neurons, in addition to hyperinflammatory and hypercoagulable states leading to increased risk of thrombotic events. These could lead to infiltration of blood-derived substances and leukocytes to the brain parenchyma [8].

The risk factors of long COVID symptoms have not been extensively studied. In some studies, certain factors that increase the risk of COVID-19 do not seem to increase the risk of long COVID [8]. For instance, although the male sex has been found to be a risk factor for contracting COVID-19, the ONS reported that the prevalence of any long COVID symptoms is higher in women than that in men (23.6% vs 20.7%) [36]. This observation is consistent with our findings where the female gender was associated with a significantly higher risk of severe long COVID symptoms.

Across different regions, studies have shown that particular occupational groups may be at higher risk of contracting COVID-19, which in turn may affect the risk of long COVID symptoms. High-risk occupations included those engaged in health care, public service, public transportation, material moving, elementary services, and other essential sectors with workers reporting higher rates of mortality. A study conducted across 46 states in the United States found that the per capita age-standardized mortality rate among essential workers was 30.4 per 100,000 individuals compared to workers in nonessential industries (15.5 per 100,000 individuals) [38]. As the risk of long COVID symptoms was associated with employment in transportation, logistics, and the discipline workforce in this study, this could be attributed to increased exposure to SARS-CoV-2, leading to a higher likelihood of experiencing persisting symptoms.

Living status, which was also regarded as an environmental risk factor, has also been examined by other studies on COVID-19 infection and long COVID symptoms. Despite a poor living environment, such as low air quality and transportation insecurity, environmental exposure to social contact that was affected by sociodemographic characteristics is also associated with the risk of COVID-19 infection. This might increase the risk of suffering from long COVID symptoms. These exposures include cohabitation and living in overcrowded housing [18]. There is a lack of studies that investigate the relationship between COVID-19 infection or long COVID symptoms and the living status of domestic workers, while our study showed that it is a significant risk factor. This may be due to environmental exposure to social contact.

Previous meta-analyses have demonstrated an increased risk of severe COVID-19 and death in current and former smokers compared to nonsmokers [39,40]. However, smoking is not a confirmed risk factor for long COVID symptoms and has only been shown to be a predictor (*P*>.001) of longer symptom duration [41]. Our findings also demonstrated that smoking prevalence is associated with a higher risk of long COVID symptoms. Smoking may potentially be a risk factor for some long COVID symptoms as smokers tend to have a greatly weakened immune and cardiovascular system, thus increasing their susceptibility to various health complications.

Multimorbidity with pre-existing health conditions [42], particularly asthma [43], has displayed a heightened risk of developing persistent symptoms and increased risk of COVID-19 fatality [44]. Multimorbidity has been positively associated with medication use, with 50% of adults in high-income countries taking up to 5 medications or more [45,46]. Poorer perceived health status may be considered a risk factor for long COVID symptoms, as observed in a study wherein 87% of the participants had a good self-reported health status before the pandemic but 83.3% reported moderate-to-poor self-reported health 6 months after initial onset [24]. This is further supported by a cohort study conducted in the Netherlands 3 months following recovery, where the health status of patients with COVID-19 was generally reported to be poor, with significant impairment in the domains of functional impairment (64%), fatigue (69%), and quality of life (72%) [47].

COVID-19 vaccination, including Coronavac-Sinovac and Pfizer-BioNTech, had protective effect on the development of long COVID symptoms. A community-based study in the United Kingdom that included 6729 patients with any severity of long COVID symptoms found that vaccine doses are associated with significant reduction in experiencing long COVID symptoms (first vaccine dose: 12.8% decrease, *P*<.001; second vaccine dose: 8.8% decrease, *P*=.003) [48]. Vaccination is able to prevent reinfection among patients with long COVID symptoms, as persons who take a single vaccine dose have equal or higher antibody titers than those who do not take any vaccination, which is consistent with our study findings [25]. The impact of vaccination on long COVID symptoms could vary among respondents or between different vaccine types; however, the vaccinated population does have a lower chance of suffering from long COVID symptoms compared with those who have not been vaccinated [25,48,49].

### Strengths and Limitations

This study was relatively large scale as compared to other surveys, and it adopted a representative sampling strategy involving 4 major cities in China. The response rate was high relative to other similar studies, thus enhancing its generalizability to other settings. Nevertheless, several limitations should be addressed. First, its cross-sectional nature did not allow a cause-and-effect relationship to be established due to the possibility of reverse causality. However, the objective of this study was to identify independent risk factors instead of causes of long COVID. In addition, there existed potential recall bias as some study participants self-reported the presence of long COVID symptoms, especially those with an earlier diagnosis of COVID-19. It should also be noted that the definition of long COVID is different across various authorities, with the National Institute for Health and Care Excellence (NICE) and the US Centers for Disease Control and Prevention (CDC) using 4-12 weeks and 4 weeks, respectively, as the time frame of its persistent symptoms. Furthermore, there could be residual confounders in our multivariate regression analysis, and these could vary according to different cities where the management strategies for COVID-19 might be different. In particular, it is unknown whether the study participants received certain treatments to relieve their long COVID symptoms at the initial stage of the development, thus reducing their risk of long COVID symptoms. Lastly, as in most surveys, not all potential study participants in the sampling frame were eligible before the recruitment process. In addition, the older population who may have a substantially higher burden of both COVID-19 and long COVID symptoms were underrepresented in this study. As the survey had to be completed through e-devices, relatively fewer older people were included as a higher proportion of them did not know how to use e-devices. Future study on the prevalence of long COVID in the older population is warranted.

### Conclusion

We examined the prevalence of long COVID symptoms in 4 Chinese cities and the effect of the severity of COVID-19. We also examined the pattern of long COVID symptoms, as well as the risk factors, including gender, occupational groups, living status, lifestyle, multimorbidity, and vaccination status. Fatigue, cough, sore throat, difficulty in concentration, feeling of anxiety, myalgia, and arthralgia are the most common severe long COVID symptoms. The female gender; engagement in transportation, logistics, or the discipline workforce; living with domestic workers; smoking; poor self-perceived health status; chronic diseases; chronic medication use; and critical severity of COVID-19 are associated with severe long COVID. Prior vaccination with ≥2 doses of COVID-19 vaccines is a protective factor. Our findings may inform early identification of patients with COVID-19 at risk of long COVID and planning of rehabilitative services.

## Appendix

Multimedia Appendix 1 Supplementary tables and figures.

## Abbreviations

aOR: adjusted odds ratio
ICU: intensive care unit
IMV: invasive mechanical ventilation
ONS: Office for National Statistics
WHO: World Health Organization

## Data Availability

The data sets generated and analyzed during this study are available from the corresponding author upon reasonable request.
