# Supplementary material for: Epidemiology, Symptomatology, and Risk Factors for Long COVID Symptoms: Population-Based, Multicenter Study
Source: JMIR Public Health Surveill. 2023 Mar 7;9:e42315. doi: 10.2196/42315 (PMC9994465; doi:10.2196/42315)
Supplement: Multimedia Appendix 1 [file publichealth_v9i1e42315_app1.docx]

**Multimedia Appendix 1. Supplementary tables and figures.**

**eSupplementary legends**

**eTable 1** Severity of COVID-19 and vaccination status of the study participants (N=2,712)

**eTable 2** Distribution of long COVID symptoms

**eFigure1** The prevalence of long COVID, moderate or severe long COVID, and severe long COVID according to time periods

**eTable 1 Severity of COVID-19 and vaccination status of the study participants (N=2,712)**

|  | **n** | **%** |
| --- | --- | --- |
| **Pneumonia at the time of diagnosis** |  |  |
| Yes | 2141 | 78.9% |
| No | 571 | 21.1% |
|  |  |  |
| **Hospital admission due to COVID-19** |  |  |
| Yes | 1944 | 71.7% |
| No | 768 | 28.3% |
|  |  |  |
| **Use of oxygen due to COVID-19** |  |  |
| Yes | 1427 | 52.6% |
| No | 1285 | 47.4% |
|  |  |  |
| **Artificial ventilation needed?** |  |  |
| Yes | 986 | 36.4% |
| No | 1726 | 63.6% |
|  |  |  |
| **Admission to Intensive Care Unit (ICU)** |  |  |
| Yes | 940 | 34.7% |
| No | 1772 | 65.3% |
|  |  |  |
| **Use of antiviral agents** |  |  |
| Yes | 1747 | 64.4% |
| No | 965 | 35.6% |
|  |  |  |
| **Severity of COVID-19** |  |  |
| Mild | 355 | 13.1% |
| Moderate | 805 | 29.7% |
| Severe | 442 | 16.3% |
| Critical | 1110 | 40.9% |
|  |  |  |
| **Vaccination status at the time of survey** |  |  |
| No | 45 | 1.7% |
| Coronavac-Sinovac, 1 dose | 101 | 3.7% |
| Coronavac-Sinovac, 2 doses | 786 | 29.0% |
| Coronavac-Sinovac, ≥ 3 doses | 1525 | 56.2% |
| Pfizer-BioNTech, 1 dose | 17 | 0.6% |
| Pfizer-BioNTech, 2 doses | 79 | 2.9% |
| Pfizer-BioNTech, ≥ 3 doses | 90 | 3.3% |
| Combination/ others | 69 | 2.5% |

**eTable 2 Distribution of long COVID symptoms**

|  |  | **Impact of symptoms on activities of daily living** | | | |
| --- | --- | --- | --- | --- | --- |
| **Bodily system** | **Symptoms** | **No** | **Slight** | **Moderate** | **Severe** |
| **General** | Fatigue | 785 (28.9%) | 1013 (37.4%) | 697 (25.7%) | 217 (8.0%) |
|  | Fever | 969 (35.7%) | 1072 (39.5%) | 582 (21.5%) | 89 (3.3%) |
|  | Chills | 1059 (39.0%) | 891 (32.9%) | 630 (23.2%) | 132 (4.9%) |
|  | Inability to perform exercise | 1187 (43.8%) | 837 (30.9%) | 501 (18.5%) | 187 (6.9%) |
|  | Night sweats | 1216 (44.8%) | 830 (30.6%) | 508 (18.7%) | 158 (5.8%) |
|  | Hair loss | 1298 (47.9%) | 744 (27.4%) | 504 (18.6%) | 166 (6.1%) |
|  | Headache | 1074 (39.6%) | 882 (32.5%) | 581 (21.4%) | 175 (6.5%) |
|  | Dizziness | 1099 (40.5%) | 833 (30.7%) | 589 (21.7%) | 191 (7.0%) |
| **Cardio-respiratory** | Chest pain | 1291 (47.6%) | 776 (28.6%) | 478 (17.6%) | 167 (6.2%) |
|  | Rapid heartbeat | 1235 (45.5%) | 799 (29.5%) | 510 (18.8%) | 168 (6.2%) |
|  | Cough | 881 (32.5%) | 966 (35.6%) | 619 (22.8%) | 246 (9.1%) |
|  | Sputum | 938 (34.6%) | 976 (36.0%) | 599 (22.1%) | 199 (7.3%) |
|  | Sore throat | 939 (34.6%) | 932 (34.4%) | 660 (24.3%) | 181 (6.7%) |
|  | Runny/blocked nose | 999 (36.8%) | 955 (35.2%) | 570 (21.0%) | 188 (6.9%) |
|  | Dyspnea | 1139 (42.0%) | 830 (30.6%) | 539 (19.9%) | 204 (7.5%) |
| **Musculoskeletal** | Arthralgia | 1092 (40.3%) | 809 (29.8%) | 605 (22.3%) | 206 (7.6%) |
|  | Myalgia | 1068 (39.4%) | 833 (30.7%) | 606 (22.3%) | 205 (7.6%) |
| **Gastrointestinal** | Nausea | 1237 (45.6%) | 779 (28.7%) | 506 (18.7%) | 190 (7.0%) |
|  | Vomiting | 1268 (46.8%) | 749 (27.6%) | 538 (19.8%) | 157 (5.8%) |
|  | Diarrhea | 1244 (45.9%) | 771 (28.4%) | 505 (18.6%) | 192 (7.1%) |
|  | Abdominal pain | 1255 (46.3%) | 780 (28.8%) | 494 (18.2%) | 183 (6.7%) |
|  | Stomachache | 1304 (48.1%) | 775 (28.6%) | 465 (17.1%) | 168 (6.2%) |
| **Special senses** | Anosmia | 1244 (45.9%) | 804 (29.6%) | 493 (18.2%) | 171 (6.3%) |
|  | Loss of taste | 1221 (45.0%) | 765 (28.2%) | 543 (20.0%) | 183 (6.7%) |
|  | Blurred vision | 1281 (47.2%) | 795 (29.3%) | 488 (18.0%) | 148 (5.5%) |
| **Neuropsychiatric** | Difficulty in concentration | 1003 (37.0%) | 881 (32.5%) | 626 (23.1%) | 202 (7.4%) |
|  | Difficulty to fall into asleep | 936 (34.5%) | 984 (36.3%) | 590 (21.8%) | 202 (7.4%) |
|  | Feeling anxious | 948 (35.0%) | 947 (34.9%) | 620 (22.9%) | 197 (7.3%) |
|  | Feeling sad | 1091 (40.2%) | 870 (32.1%) | 558 (20.6%) | 193 (7.1%) |
|  | Memory problem | 1094 (40.3%) | 885 (32.6%) | 533 (19.7%) | 200 (7.4%) |
| **Other symptoms** | | 1875 (69.1%) | 439 (16.2%) | 290 (10.7%) | 108 (4.0%) |

**eFigure1 The prevalence of long COVID, moderate or severe long COVID, and severe long COVID according to time periods**
